# Isolation and characterization of Pb- and Cr-tolerant phosphate-solubilizing bacteria and optimization of culture conditions

**DOI:** 10.3389/fmicb.2025.1759460

**Published:** 2026-02-03

**Authors:** Huanhuan Jiang, Lu Chen, ShiYao Zeng, Xiangping Xu, Jieying Zhang, MinHua Liang, Jiamin Zhang, Shengnian Liang, Qianhua Ji

**Affiliations:** College of Life Sciences, Zhaoqing University, Zhaoqing, China

**Keywords:** bioremediation, chromium, culture conditions, heavy metal tolerance, lead, phosphate-solubilizing bacteria

## Abstract

**Introduction:**

Phosphate-solubilizing bacteria (PSB) that tolerate heavy metals may enhance phosphorus availability in contaminated soils and provide candidates for bio-based management.

**Methods:**

PSB were isolated from farmland rhizosphere soil and screened on tricalcium phosphate medium. Soluble phosphorus (soluble P) was quantified in liquid culture using tricalcium phosphate, ferric phosphate, aluminum phosphate, and lecithin as insoluble phosphorus (insoluble P) sources. Pb/Cr tolerance was assessed by growth on metal-amended plates and minimum inhibitory concentration (MIC) assays, and six dominant isolates were identified by 16S rRNA gene sequencing.

**Results:**

Eighteen PSB isolates were obtained. All strains solubilized tricalcium phosphate (2.82253.53 mg L^-1^) and ferric phosphate (6.24206.48 mg L^-1^); most also solubilized aluminum phosphate (13.0244.73 mg L^-1^), and 13 isolates solubilized lecithin (2.8230.84 mg L^-1^). The six strains able to grow at 6 mmol L^-1^ Pb or Cr were identified as *Bacillus* sp. (HY-1, HY-6), *Bacillus subtilis* (HY-3, HY-16), *Bacillus thuringiensis* (HY-12), and *Duganella* sp. (HY-13). MICs were higher for Cr (1720 mmol L^-1^) than for Pb (67 mmol L^-1^), and increasing Pb or Cr suppressed both growth (OD_600_) and phosphate solubilization. Single-factor optimization identified glucose as the most suitable carbon source and 0.3 g L^-1^ NaCl as optimal; the best initial pH was 6 for five strains (pH 7 for HY-12), and temperature optima were strain-dependent (3040 °C).

**Conclusion:**

These results define cultivation parameters for inoculum preparation and support further testing in soil and plant systems under environmentally relevant conditions.

## 1 Introduction

Phosphorus (P) is an essential macronutrient for plant growth, playing critical roles in cellular respiration, photosynthesis, and the metabolism of diverse nutrients (Khan et al., 2023). Plants primarily obtain P from the soil; however, more than 70% of soil P occurs in insoluble forms resulting from reactions with metal ions such as Aluminum ion (Al^3+^), Iron (III) ion (Fe^3+^) and Calcium ion (Ca^2+^), making it largely unavailable for plant uptake (López-Bucio et al., 2000). As a major global producer and consumer of phosphate fertilizers, China has long accounted for nearly 30% of global consumption, but its farmland P use efficiency remains below the global average (Wang et al., 2022). At the same time, rapid industrial and agricultural development has led to increasingly severe heavy metal contamination of agricultural soils in China. Among these pollutants, the proportions of monitoring sites exceeding the national standards for Pb and Cr have been reported as 1.5 and 1.1%, respectively (Sun et al., 2019). Lead and chromium tend to accumulate in crops, which not only reduce food quality and hander agricultural development but also pose risks to human health by entering the body through the food chain and damaging the nervous system and internal organs (Kotnala et al., 2025). In addition, heavy metals can alter the composition and diversity of soil microbial communities and reduce plant biomass, ultimately leading to the loss of soil fertility (Pérez-de-Mora et al., 2006). Conventional agricultural practices often rely on the application of chemical phosphate fertilizers to increase soil P and meet crop demand. However, long-term and excessive use of phosphate fertilizers promotes the formation of poorly soluble P precipitates, resulting in soil compaction, yield and quality decline, and exacerbation of heavy metal pollution (Liu et al., 2021). Consequently, reducing chemical fertilizer inputs, lowering soil heavy metal concentrations, and increasing available soil P have become key issues in the development of green and sustainable agriculture in China.

Phosphate-solubilizing bacteria (PSB) are a group of microorganisms capable of mobilizing insoluble or sparingly soluble P in soils. They can enhance soil fertility while simultaneously alleviating heavy metal toxicity, thereby offering an ecological approach for the remediation of heavy metal–contaminated soils (Chen and Liu, 2019). The phosphate-solubilizing mechanisms of PSB mainly involve the secretion of organic acids and phosphatases during growth, which can mobilize insoluble P compounds in soils. Moreover, heavy metal–tolerant PSB can release phosphate from insoluble pools and react with heavy metal ions to form insoluble metal phosphates, thereby immobilizing the metals and reducing their bioavailability (Sahu et al., 2022). Previous studies have primarily focused on the ability of PSB to release soil P and promote plant growth (Sundara et al., 2002; Wang et al., 2022). In recent years, the role of PSB in the immobilization of Pb and Cr in soils has attracted increasing attention. For example, Teng et al. (2019) reported that PSB can convert insoluble P into soluble forms and further react with Pb to form more stable lead phosphate minerals, such as Pb₅(PO₄)₃Cl and Pb₅(PO₄)₃. Sarkar et al. (2019) demonstrated that *Pseudomonas laurentiana* can chelate Pb, As and Cr, thereby binding heavy metals to the cell wall and reducing their biological toxicity. Oves et al. (2019) isolated strain OS2 from contaminated soils and demonstrated that it exhibited strong survival under high heavy metal concentrations, with phosphate accumulation by the strain showing a positive correlation with Cr binding. Park et al. (2011) obtained two PSB strains, *Pantoea* sp. and *Enterobacter* sp., which immobilized Pb by dissolving phosphate minerals and releasing phosphate ions. Zhao et al. (2022) showed that a combination of PSB and biochar-supported nano-hydroxyapatite effectively immobilized Cd in river sediments. To date, numerous PSB strains have been isolated from different ecological environments and crop rhizospheres and have been shown to promote plant growth, including members of the genera *Bacillus*, *Pseudomonas*, *Azotobacter*, *Enterobacter* and *Micrococcus* (Kalayu, 2019; Paul and Sinha, 2017; Mendoza-Arroyo et al., 2020). However, relatively few studies have focused on the metabolic characteristics and biological properties of PSB under heavy metal stress, and research on the optimization of growth conditions for heavy metal-tolerant PSB is even more scarce. In southern China, soil acidification and heavy metal contamination are particularly severe (Xiao et al., 2020). In the present study, rhizosphere soils were collected from farmland in Baitu Town, Gaoyao District, Zhaoqing City, Guangdong Province, China and PSB were screened using tricalcium phosphate as the sole P source. After characterizing their phosphate-solubilizing properties, we further selected strains with high Pb and Cr tolerance. We analyzed changes in growth and phosphate solubilization of PSB under Pb and Cr stress and optimized their fermentation conditions to determine the optimal culture parameters. These strains are expected to serve as microbial resources for the remediation of heavy metal–contaminated soils.

## 2 Materials and methods

### 2.1 Soil sampling

Rhizosphere soil samples were collected on December 23, 2021, from a crop field in Baitu Town, Gaoyao District, Zhaoqing City, Guangdong Province, China (23°00′N, 112°58′E). At each sampling point, surface litter around the crop roots was carefully removed, and soil samples were collected from a depth of 20 cm using a sterile soil auger. Larger soil aggregates adhering to roots and visible plant residues were gently shaken off. The collected soil was thoroughly mixed, placed into sterile sampling bags, transported to the laboratory, and stored at 4 °C and proceed with strain isolation the following day.

The physicochemical properties of the soil are summarized in Table 1.

**Table 1:** Physicochemical properties of the soil.

Total N content (mg/kg)	Total P content (mg/kg)	Total K content (mg/kg)	Temperature (°C)	Humidity (%)
21	20	55	20	50
23	24	55	20	83
20	20	55	19	50

### 2.2 Culture media

The inorganic phosphate liquid medium (TPM) contained (per liter): yeast extract 0.5 g, tricalcium phosphate 5.0 g, (NH₄)₂SO₄ 0.5 g, glucose 10.0 g, NaCl 0.3 g, MgSO₄·7H₂O 0.3 g, KCl 0.3 g, MnSO₄ 0.03 g and FeSO₄·7H₂O 0.03 g; the pH was adjusted to 7.2 and the volume made up to 1,000 mL. To determine the solubilization of other inorganic phosphates, tricalcium phosphate was replaced by 5 g/L ferric phosphate or aluminum phosphate. For the organic phosphate medium (YM), tricalcium phosphate was replaced by egg yolk as the organic P source. Solid media were prepared by adding 20 g/L agar to the corresponding liquid media.

### 2.3 Isolation and purification of PSB

Ten grams of soil were placed in a 250-mL Erlenmeyer flask containing 90 mL sterile water and shaken on a shaking incubator at constant temperature for 30 min to fully disperse the soil particles. One milliliter of the supernatant was serially diluted 10^−1^ to 10^−5^. Aliquots (0.1 mL) of the 10^−3^–10^−5^ dilutions were spread onto YM and TPM agar plates and incubated in an inverted position at 30 °C for 3 days. Colonies with distinct morphology and/or visible phosphate-solubilizing halos were picked and streaked repeatedly (4–5 rounds) for purification. Purified isolates were transferred onto beef extract–peptone slants and stored at 4 °C for subsequent experiments.

### 2.4 Determination of phosphate-solubilizing capacity

Each PSB isolate was inoculated into liquid media containing different insoluble P sources at an inoculum size of 1% (v/v) and incubated on a shaking incubator at 30 °C and 180 r/min for 3 days. Uninoculated media served as blanks. Each treatment consisted of three biological replicates. A 5 mg/L P standard stock solution was prepared to construct a standard curve. After incubation, cultures were centrifuged at 1200 rpm (16 × g) for 10 min and the soluble P concentration in the supernatant was determined using the molybdenum–antimony colorimetric method (Kitson and Mellon, 1944).

### 2.5 Analysis of lead and chromium tolerance

To evaluate heavy metal tolerance, 0.1 mL of bacterial suspension was spread onto beef extract–peptone agar plates supplemented with Pb or Cr at concentrations of 0, 2, 4 and 6 mmol/L. Plates were incubated at 28 °C for 4 days, and bacterial growth was examined. Isolates that grew on plates containing 6 mmol/L Pb or Cr were regarded as heavy metal–tolerant strains. Before assessing the effects of different Pb and Cr concentrations on phosphate-solubilizing activity, we determined the minimal inhibitory concentration (MIC) of Pb and Cr for each of the six selected PSB strains. Briefly, 0.1 mL of bacterial suspension was spread onto beef extract–peptone agar plates supplemented with increasing concentrations of Pb or Cr and incubated at 28 °C for 4 d. The highest metal concentration at which no visible growth, defined as the absence of colonies detectable by the naked eye, was recorded as the MIC. Strains with relatively high MIC values were considered more tolerant to heavy metals and were used for subsequent experiments.

### 2.6 Effects of Pb and Cr on growth and metabolism of PSB

The six selected PSB strains were inoculated at 1% (v/v) into TPM media containing different concentrations of Pb or Cr. For each strain and metal treatment, three replicate flasks were prepared, and uninoculated media with the same metal concentration served as blanks. Cultures were incubated at 30 °C and 180 r/min for 3 days. Bacterial growth was monitored by measuring optical density at 600 nm (OD₆₀₀), and soluble P concentrations in the supernatants were determined using the molybdenum–antimony colorimetric method. Both measurements were performed after 3 d of incubation.

### 2.7 Optimization of fermentation conditions for lead- and chromium-tolerant PSB

Using TPM as the basal medium, single-factor experiments were performed to optimize the culture conditions of the six heavy metal–tolerant PSB strains. The tested factors included carbon source (glucose, fructose, sucrose, starch, lactose, and mannitol), with each sugar added at a concentration of 5 g/L, temperature (20, 25, 30, 35, and 40 °C), initial pH (5.0, 6.0, 7.0, 8.0, and 9.0) and NaCl concentration (0.1, 0.3, 0.5, 0.7, and 0.9 g/L). For each factor, only one condition was varied at a time while the others were kept constant. Cultures were incubated at 30 °C and 180 r/min for 3 days. After incubation, OD₆₀₀ and soluble phosphate concentrations were measured. All experimental data were statistically analyzed via one-way analysis of variance (ANOVA) with Duncan’s multiple comparison test using SPSS Statistics software, and differences were deemed significant at *p* < 0.05.

### 2.8 Molecular identification of PSB

Genomic DNA of the six PSB strains (HY-1, HY-3, HY-6, HY-12, HY-13 and HY-16) was extracted using a commercial bacterial genomic DNA extraction kit (Vazyme Biotech Co., Ltd., Nanjing, China) according to the manufacturer’s instructions. The nearly full-length 16S rDNA region was amplified using universal bacterial primers 27F (5’-AGAGTTTGATCCTGGCTCAG-3′) and 1492R (5’-GGTTACCTTGTTACGACTT-3′). PCR products were sequenced by Sangon Biotech (Shanghai) Co., Ltd. The resulting sequences were submitted to the NCBI database for BLAST analysis, and closely related sequences with a similarity threshold of ≥97% were downloaded for subsequent phylogenetic analysis. Multiple sequence alignment was performed using Clustal X, and a phylogenetic tree was constructed by the neighbor-joining method in MEGA 7.0 to determine the taxonomic affiliation of the PSB strains.

## 3 Results

### 3.1 Isolation of PSB and determination of phosphate-solubilizing capacity

Using the plate spread method, 18 PSB strains were isolated and purified from the rhizosphere soil and designated HY-1 through HY-18. Their abilities to solubilize different P sources were evaluated using tricalcium phosphate, aluminum phosphate and ferric phosphate as sparingly soluble inorganic phosphate sources and lecithin as an organic phosphate source (Table 2). All 18 PSB strains solubilized both tricalcium phosphate and ferric phosphate. a significant difference was detected in their solubilizing efficiency, With soluble phosphate concentrations ranging from 2.82 to 253.53 mg/L. Eight strains exhibited tricalcium phosphate–solubilizing capacities above 40 mg/L, and strain HY-12 showed the highest activity, reaching 253.53 mg/L. The ferric phosphate–solubilizing capacities of the isolates ranged from 6.24 to 206.48 mg/L. Most strains also solubilized aluminum phosphate, with soluble phosphate concentrations ranging from 13.02 to 44.73 mg/L. Thirteen strains solubilized lecithin, with soluble phosphate concentrations between 2.82 and 30.84 mg/L. Overall, the phosphate-solubilizing capacities of the isolates for different P sources followed the order: tricalcium phosphate > ferric phosphate > aluminum phosphate > lecithin.

**Table 2:** Phosphate solubilizing ability of different insoluble phosphorus sources by the PSB.

Strain number	The content of soluble phosphorus (mg/L)			
	Inorganic phosphorus	Ferric phosphate	Aluminum phosphate	Lecithin
HY-1	44.22 ± 1.16f	16.70 ± 0.76h	14.93 ± 1.81h	22.34 ± 0.29b
HY-2	11.25 ± 0.10jk	6.24 ± 0.50l	39.08 ± 0.61b	—
HY-3	40.61 ± 0.22g	82.26 ± 0.48de	13.02 ± 0.55i	4.34 ± 0.19h
HY-4	13.09 ± 0.19j	24.44 ± 0.11g	15.43 ± 0.79h	2.82 ± 0.19i
HY-5	17.78 ± 0.29i	10.87 ± 0.44k	35.79 ± 0.40c	—
HY-6	86.83 ± 0.67b	115.43 ± 0.57b	19.05 ± 0.55f	15.62 ± 0.29d
HY-7	10.99 ± 0.66jk	22.09 ± 0.29g	—	12.45 ± 0.67e
HY-8	6.05 ± 0.19mn	13.21 ± 0.72ijk	32.17 ± 0.29e	—
HY-9	8.20 ± 0.11lm	11.12 ± 0.40jk	13.15 ± 0.11i	4.21 ± 0.22h
HY-10	9.28 ± 0.00kl	15.24 ± 0.29hi	34.65 ± 0.22d	6.56 ± 0.22fg
HY-11	48.98 ± 0.22e	21.58 ± 0.29g	17.53 ± 0.79g	6.11 ± 0.22g
HY-12	253.53 ± 1.51a	94.88 ± 5.27c	44.73 ± 0.77a	—
HY-13	76.81 ± 1.49d	83.91 ± 1.08d	39.53 ± 0.19b	12.20 ± 0.29e
HY-14	48.66 ± 0.33e	80.36 ± 2.42e	36.74 ± 0.11c	—
HY-15	5.10 ± 0.19n	14.10 ± 0.22hij	—	30.84 ± 0.48a
HY-16	80.05 ± 3.13c	206.48 ± 0.86a	15.12 ± 0.22h	7.00 ± 0.50f
HY-17	35.91 ± 2.20h	16.26 ± 0.22 h	38.89 ± 0.22b	15.94 ± 0.19d
HY-18	5.42 ± 0.61n	70.22 ± 0.29f	34.65 ± 0.22d	21.52 ± 0.72c

Different letters in the table indicate significant differences among various strains at the *p* < 0.05 level.

### 3.2 Lead and chromium tolerance of PSB

**Figure 1:**
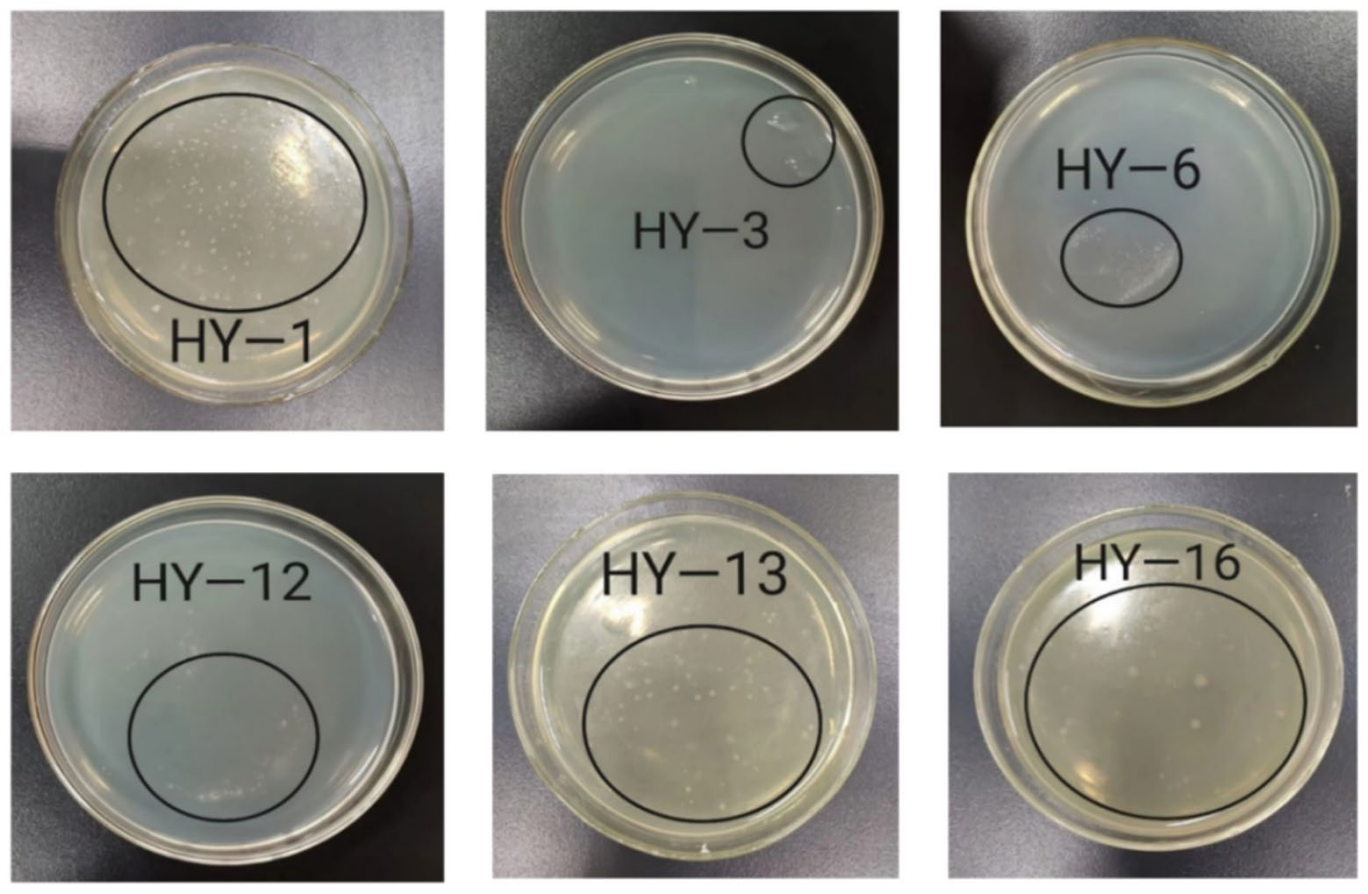
Growth of six lead- and chromium-tolerant PSB on beef extract–peptone agar supplemented with 6 mmol L^−1^ heavy metal (Pb or Cr) after 4 d incubation at 28 °C. Plates show representative colonies of strains HY-1, HY-3, HY-6, HY-12, HY-13 and HY-16, with the circled areas indicating typical growth used to assess metal tolerance.

Heavy metal tolerance was assessed by spreading 0.1 mL bacterial suspension onto beef extract–peptone agar plates with 0, 2, 4, or 6 mmol/L Pb/Cr, followed by incubation at 28 °C for 4 days. Strains growing on 6 mmol/L Pb/Cr plates were deemed tolerant. As shown in Figure 1, only six PSB strains (HY-1, HY-3, HY-6, HY-12, HY-13, and HY-16) exhibited growth on these plates, confirming their tolerance.

**Figure 2:**
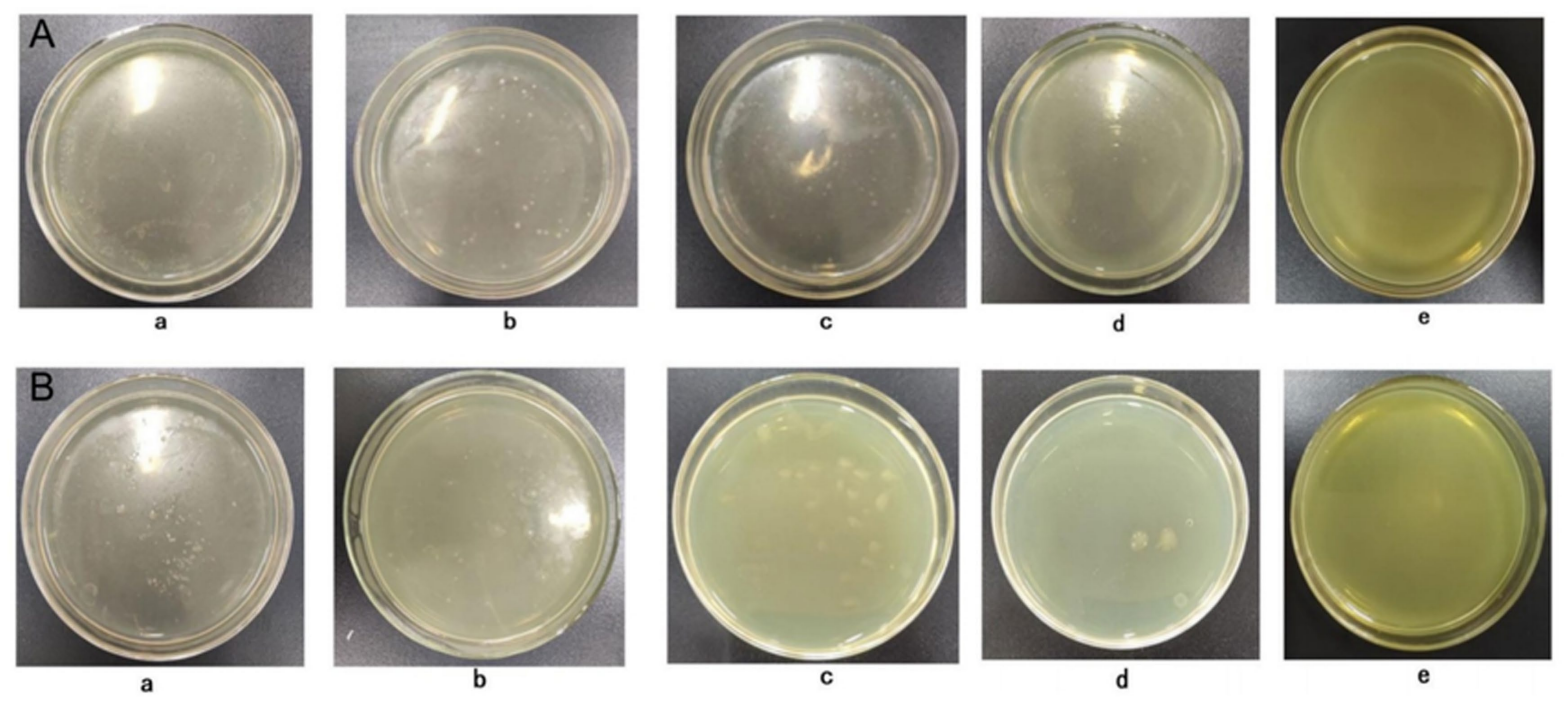
Determination of the minimal inhibitory concentration (MIC) of chromium for representative PSB strains. **(A)** Colony growth of a representative PSB strain on beef extract–peptone agar containing increasing concentrations of Cr (a–e, from 0 up to the highest concentration tested) after 4 d of incubation at 28 °C. **(B)** Colony growth of another PSB strain under the same conditions; disappearance of colonies on plate e indicates the MIC.

To further determine the minimum metal concentrations inhibiting the growth of these six strains, MIC values were determined (Figure 2 shows the MIC results for Cr/Pb of HY-13 as an example). The MIC values for each strain were as follows: HY-1, 20 mmol/L for Cr and 7 mmol/L for Pb; HY-3, 18 mmol/L for Cr and 7 mmol/L for Pb; HY-6, 19 mmol/L for Cr and 6 mmol/L for Pb; HY-12, 18 mmol/L for Cr and 6 mmol/L for Pb; HY-13, 17 mmol/L for Cr and 7 mmol/L for Pb; and HY-16, 18 mmol/L for Cr and 7 mmol/L for Pb. All six strains therefore exhibited relatively high Pb and Cr tolerance and strong environmental adaptability, indicating promising potential for the bioremediation of heavy metal–contaminated soils.

### 3.3 Effects of Pb and Cr stress on growth and phosphate-solubilizing activity

**Figure 3:**
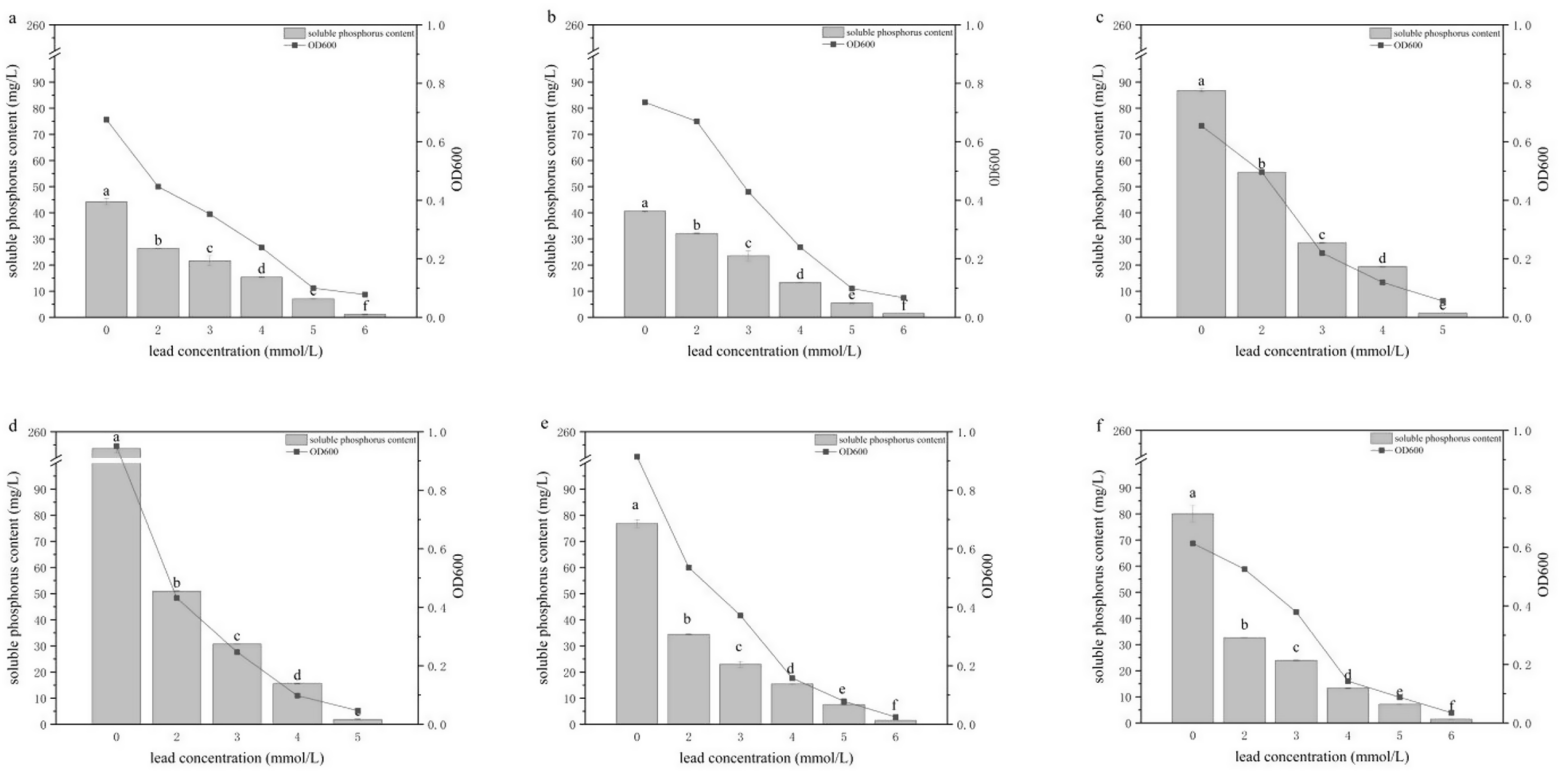
Effects of different Pb concentrations on the growth and phosphate-solubilizing activity of PSB strains HY-1 **(a)**, HY-3 **(b)**, HY-6 **(c)**, HY-12 **(d)**, HY-13 **(e)** and HY-16 **(f)** in TPM medium. Bars indicate soluble P content, and lines indicate cell growth (OD₆₀₀) after 3 d of incubation at 30 °C. Data are means ± SD (n = 3). Bars with different lowercase letters indicate statistically significant differences among carbon sources for each strain (*p* < 0.05).

**Figure 4:**
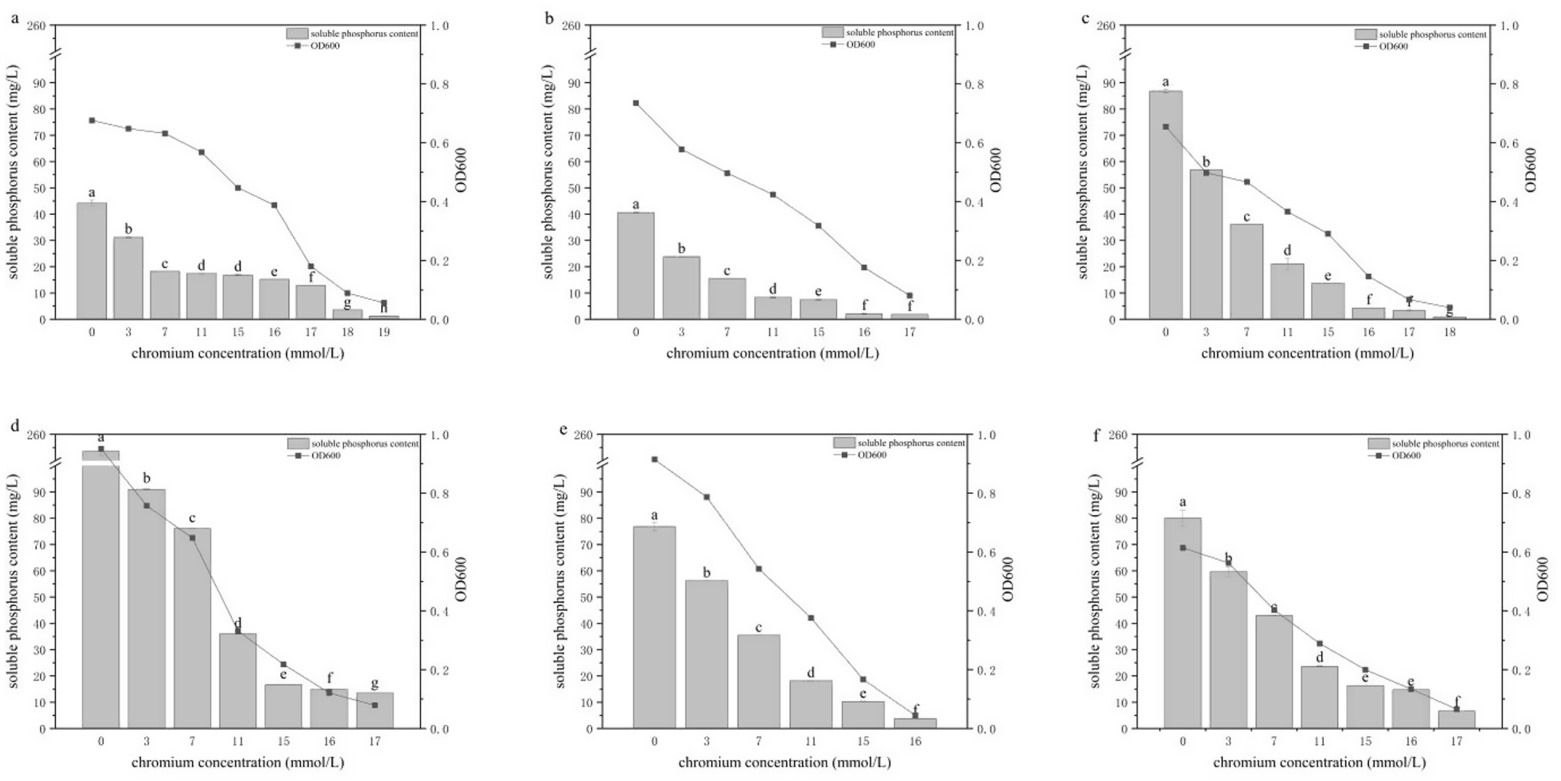
Effects of different Cr concentrations on the growth and phosphate-solubilizing activity of PSB strains HY-1 **(a)**, HY-3 **(b)**, HY-6 **(c)**, HY-12 **(d)**, HY-13 **(e)** and HY-16 **(f)** in TPM medium. Bars represent soluble phosphorus content, and lines represent cell density (OD₆₀₀) after 3 d of incubation at 30 °C. Data are means ± SD (*n* = 3). Bars with different lowercase letters indicate statistically significant differences among carbon sources for each strain (*p* < 0.05).

The growth (OD₆₀₀) and soluble phosphate concentrations of the six PSB strains were measured after 3 days of cultivation in TPM media containing different concentrations of Pb or Cr (Figures 3, 4). Under Cr stress, the upper tolerance limits of the six strains differed. HY-1 showed the highest tolerance and ceased growth only at 20 mmol/L Cr, whereas the tolerance limits of HY-3, HY-12 and HY-16 were 18 mmol/L, HY-6 was inhibited at 19 mmol/L, and HY-13 had the lowest tolerance, with growth inhibited at 17 mmol/L. At Cr concentrations of 3, 7 and 11 mmol/L, both growth and phosphate solubilization decreased with increasing Cr levels. The OD₆₀₀ values ranged from 0.289 to 0.787, with HY-13 showing the highest OD₆₀₀ (0.787) at 3 mmol/L Cr and HY-16 showing the lowest (0.289) at 11 mmol/L Cr. Soluble phosphate concentrations ranged from 8.33 to 91.02 mg/L; HY-12 displayed the highest phosphate-solubilizing activity (91.02 mg/L) at 3 mmol/L Cr, whereas HY-3 showed the lowest (8.33 mg/L) at 11 mmol/L Cr. Overall, the six PSB strains were sensitive to elevated Pb and Cr concentrations. Their growth and phosphate-solubilizing activities were inhibited under metal stress, and the degree of inhibition increased markedly with increasing metal concentration. Both OD₆₀₀ and soluble phosphate concentrations were negatively correlated with Pb and Cr levels.

### 3.4 Effects of carbon source on phosphate solubilization

**Figure 5:**
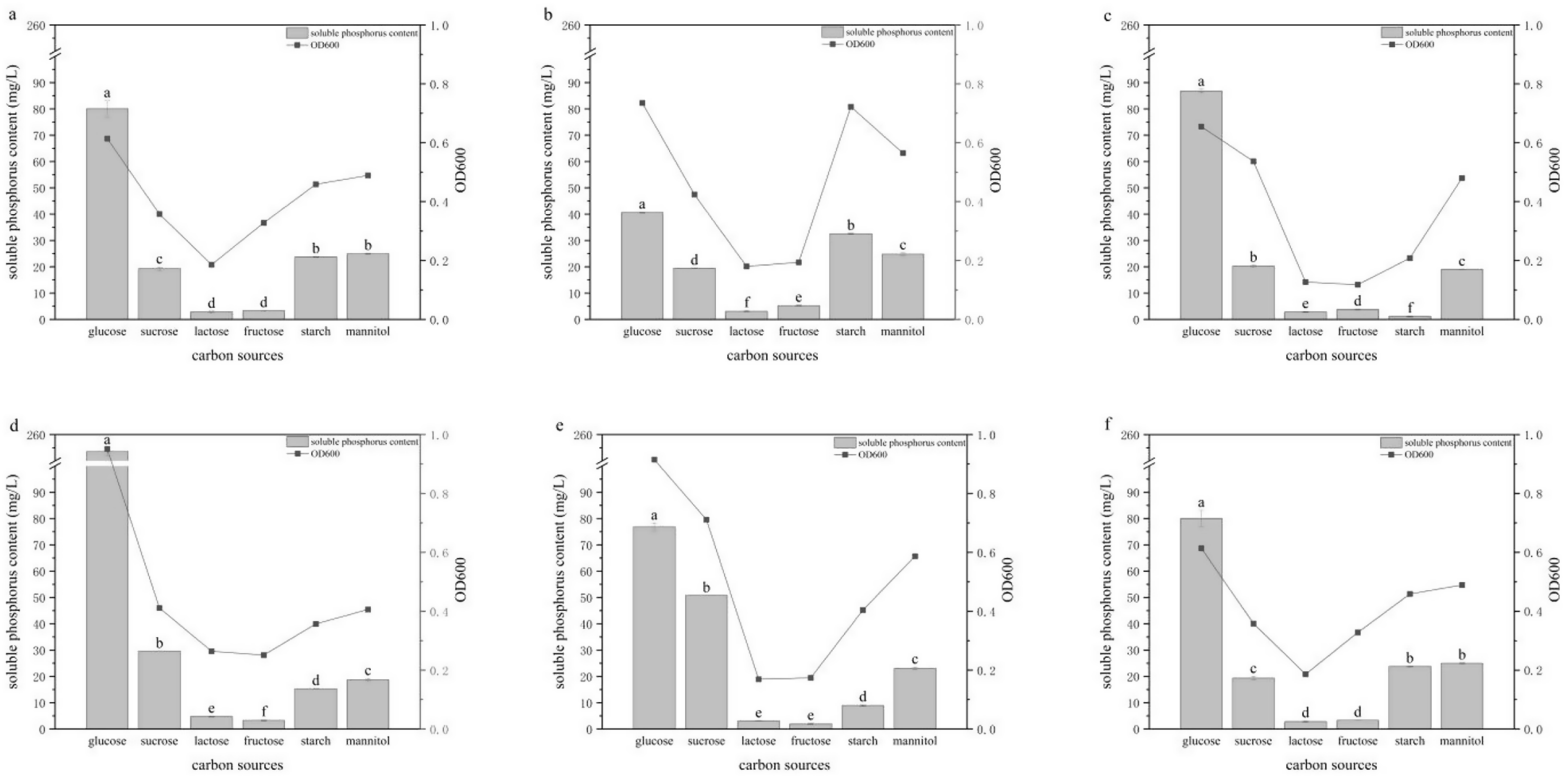
Effects of different carbon sources on the growth and phosphate-solubilizing activity of PSB strains HY-1 **(a)**, HY-3 **(b)**, HY-6 **(c)**, HY-12 **(d)**, HY-13 **(e)** and HY-16 **(f)** in TPM medium. Glucose, starch, lactose, fructose, sucrose and mannitol were used as sole carbon sources. Bars represent soluble P content, and lines represent cell density (OD₆₀₀) after 3 d of incubation at 30 °C and 180 r/min. Bars with different lowercase letters indicate statistically significant differences among carbon sources for each strain (*p* < 0.05).

To determine the optimal carbon source for the six PSB strains (HY-1, HY-3, HY-6, HY-12, HY-13, and HY-16), six different carbon sources—glucose, fructose, sucrose, starch, lactose and mannitol—were tested (Figure 5). Growth and soluble phosphate concentrations were used to assess carbon source preference. Glucose was the most favorable carbon source for all strains, supporting the best growth and highest phosphate-solubilizing activity. Under glucose supplementation, the soluble phosphate concentrations of HY-1, HY-3, HY-6, HY-12, HY-13 and HY-16 reached 44.22, 40.61, 86.83, 253.53, 76.81 and 80.05 mg/L, respectively, with HY-12 showing the most pronounced effect. In contrast, lactose strongly inhibited both growth and phosphate solubilization in all strains, with soluble phosphate concentrations of only 2.82–4.72 mg/L, making it the least favorable carbon source. The six strains differed in their utilization of the other four carbon sources. HY-1 and HY-3 showed a preference of starch > sucrose > mannitol > fructose; HY-6, HY-12 and HY-13 preferred sucrose and mannitol; and HY-16 favored mannitol over starch. Nevertheless, phosphate-solubilizing activity on non-glucose carbon sources was consistently and markedly lower than that observed with glucose.

### 3.5 Effects of temperature on phosphate solubilization

**Figure 6:**
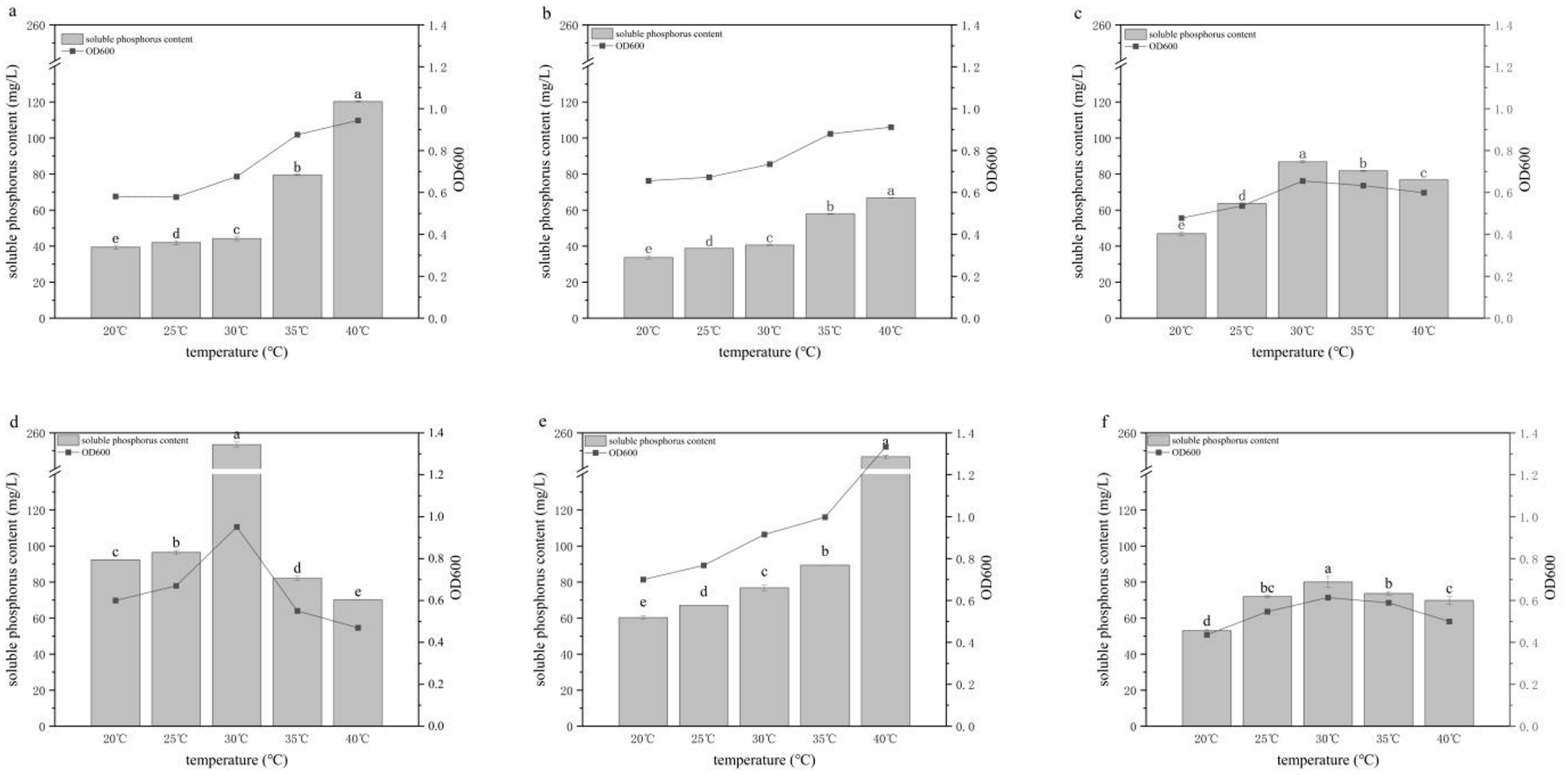
Effects of temperature on the growth and phosphate-solubilizing activity of PSB strains HY-1 **(a)**, HY-3 **(b)**, HY-6 **(c)**, HY-12 **(d)**, HY-13 **(e)** and HY-16 **(f)** in TPM medium with glucose as the sole carbon source. Bars represent soluble P content, and lines represent cell density (OD₆₀₀) after 3 d of incubation at the indicated temperatures (20–40 °C, 180 r/min). Data are means ± SD (*n* = 3). Different lowercase letters above the bars indicate significant differences among temperatures for each strain (*p* < 0.05).

Using glucose as the sole carbon source, the temperature tolerance and optimal growth temperature of the six PSB strains were investigated at temperatures from 20 to 40 °C (Figure 6). All strains were able to grow across this temperature range, indicating broad temperature tolerance, but their phosphate-solubilizing capacities varied substantially. For HY-1, HY-3 and HY-13, both OD₆₀₀ and soluble phosphate concentrations increased with temperature, reaching maxima at 40 °C, with phosphate concentrations of 120.25, 66.79 and 246.74 mg/L, respectively, indicating a degree of thermotolerance. In contrast, HY-6, HY-12 and HY-16 exhibited optimal growth and phosphate solubilization at 30 °C, achieving soluble phosphate concentrations of 86.83, 253.53 and 80.05 mg/L, respectively. When the temperature exceeded 30 °C, growth and phosphate-solubilizing capacities of these strains decreased, with HY-12 being the most strongly affected.

### 3.6 Effects of pH on phosphate solubilization

**Figure 7:**
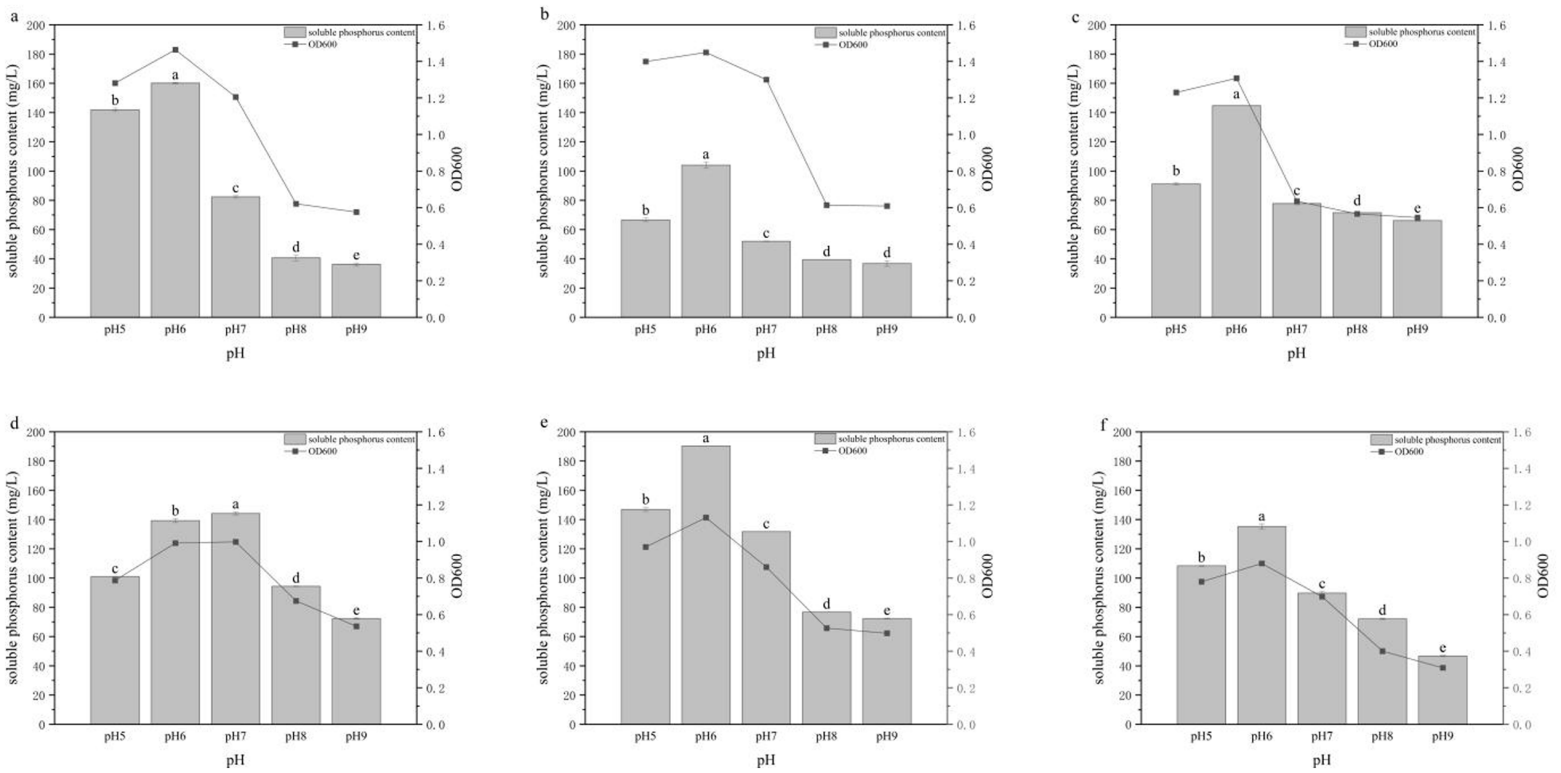
Effects of initial pH on the growth and phosphate-solubilizing activity of PSB strains HY-1 **(a)**, HY-3 **(b)**, HY-6 **(c)**, HY-12 **(d)**, HY-13 **(e)** and HY-16 **(f)** in TPM medium with glucose as the sole carbon source. Bars represent soluble P content, and lines represent cell density (OD₆₀₀) after 3 d of incubation at 30 °C and 180 r/min. Data are means ± SD (*n* = 3). Different lowercase letters above the bars indicate significant differences among pH treatments for each strain (*p* < 0.05).

The pH tolerance range and optimal pH for growth of the six PSB strains are shown in Figure 7. All strains grew over a pH range of 5–9, confirming a relatively broad pH tolerance. However, phosphate-solubilizing capacities differed significantly among pH treatments. Strains HY-1, HY-3, HY-6, HY-13 and HY-16 exhibited better growth and higher soluble phosphate concentrations under mildly acidic conditions, with both parameters peaking at pH 6. At this pH, the phosphate-solubilizing capacities of these strains were 160.13, 104.08, 144.72, 190.31 and 135.15 mg/L, respectively. In contrast, HY-12 showed the highest phosphate-solubilizing activity at neutral pH (pH 7), with a soluble phosphate concentration of 144.21 mg/L. At higher pH values, growth of all strains was inhibited and their phosphate-solubilizing capacities declined. These results indicate that the optimal pH for HY-1, HY-3, HY-6, HY-13 and HY-16 is 6, whereas HY-12 exhibits an optimal pH of 7.

### 3.7 Effects of NaCl concentration on phosphate solubilization

**Figure 8:**
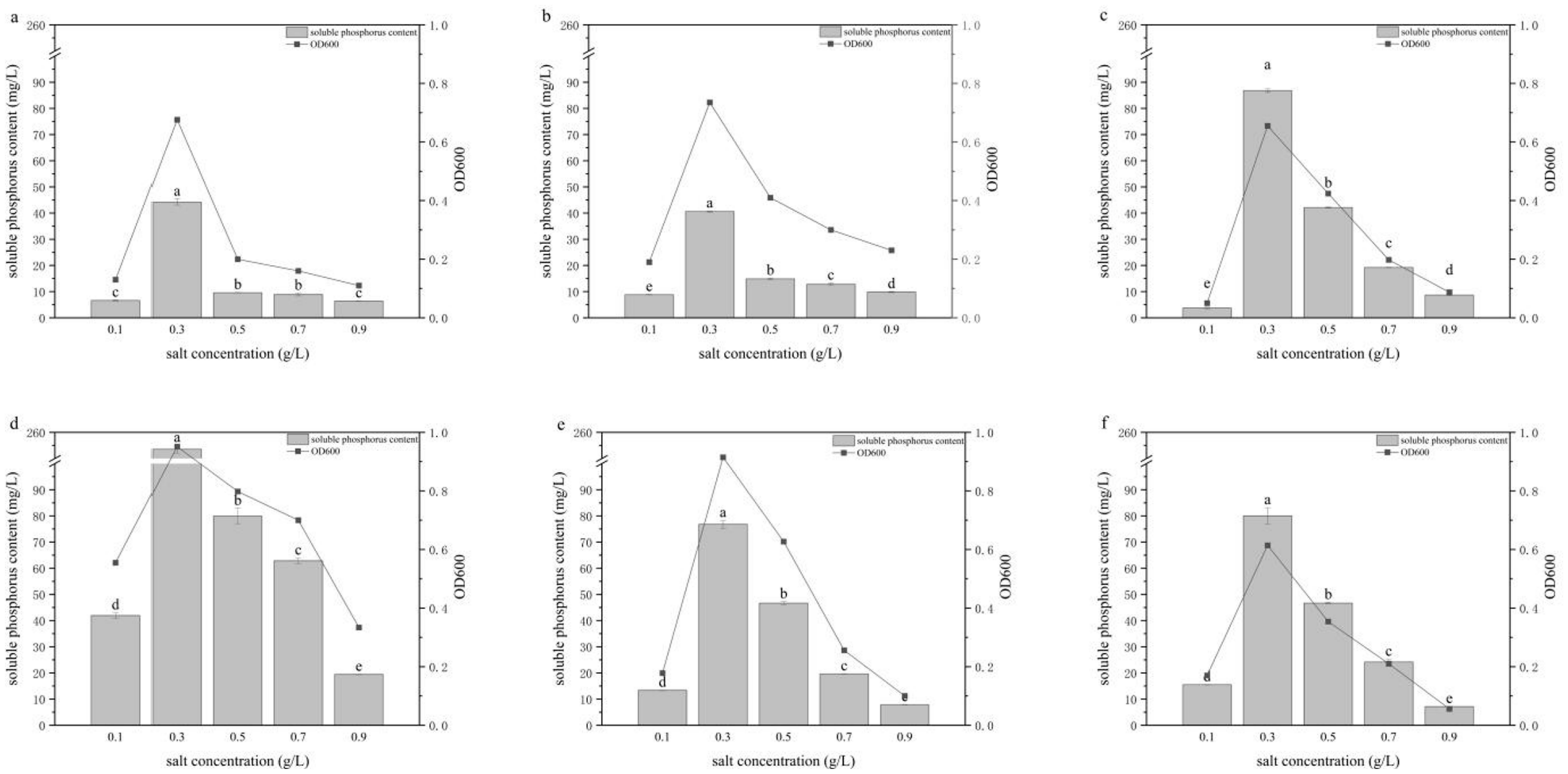
Effects of NaCl concentration on the growth and phosphate-solubilizing activity of PSB strains HY-1 **(a)**, HY-3 **(b)**, HY-6 **(c)**, HY-12 **(d)**, HY-13 **(e)** and HY-16 **(f)** in TPM medium with glucose as the sole carbon source. Bars represent soluble P content, and lines represent cell density (OD₆₀₀) after 3 d of incubation at 30 °C and 180 r/min under different NaCl concentrations (0.1–0.9 g L^−1^). Data are means ± SD (*n* = 3). Different lowercase letters above the bars indicate significant differences among salt treatments for each strain (*p* < 0.05).

The NaCl concentration affects the osmotic pressure of the culture medium and thereby influences bacterial growth and metabolism. To investigate the effects of osmotic stress on the six PSB strains, NaCl concentrations of 0.1, 0.3, 0.5, 0.7 and 0.9 g/L were tested (Figure 8). All strains grew across this NaCl gradient, but their phosphate-solubilizing capacities varied significantly. At 0.3 g/L NaCl, growth was best and soluble phosphate concentrations were highest, with phosphate-solubilizing capacities of 44.22, 40.60, 86.83, 253.53, 76.81 and 80.05 mg/L for HY-1, HY-3, HY-6, HY-12, HY-13 and HY-16, respectively. All six strains tolerated NaCl concentrations up to 0.9 g/L, indicating effective osmoregulatory capacity and a certain degree of salt tolerance.

### 3.8 Molecular identification of PSB

**Figure 9:**
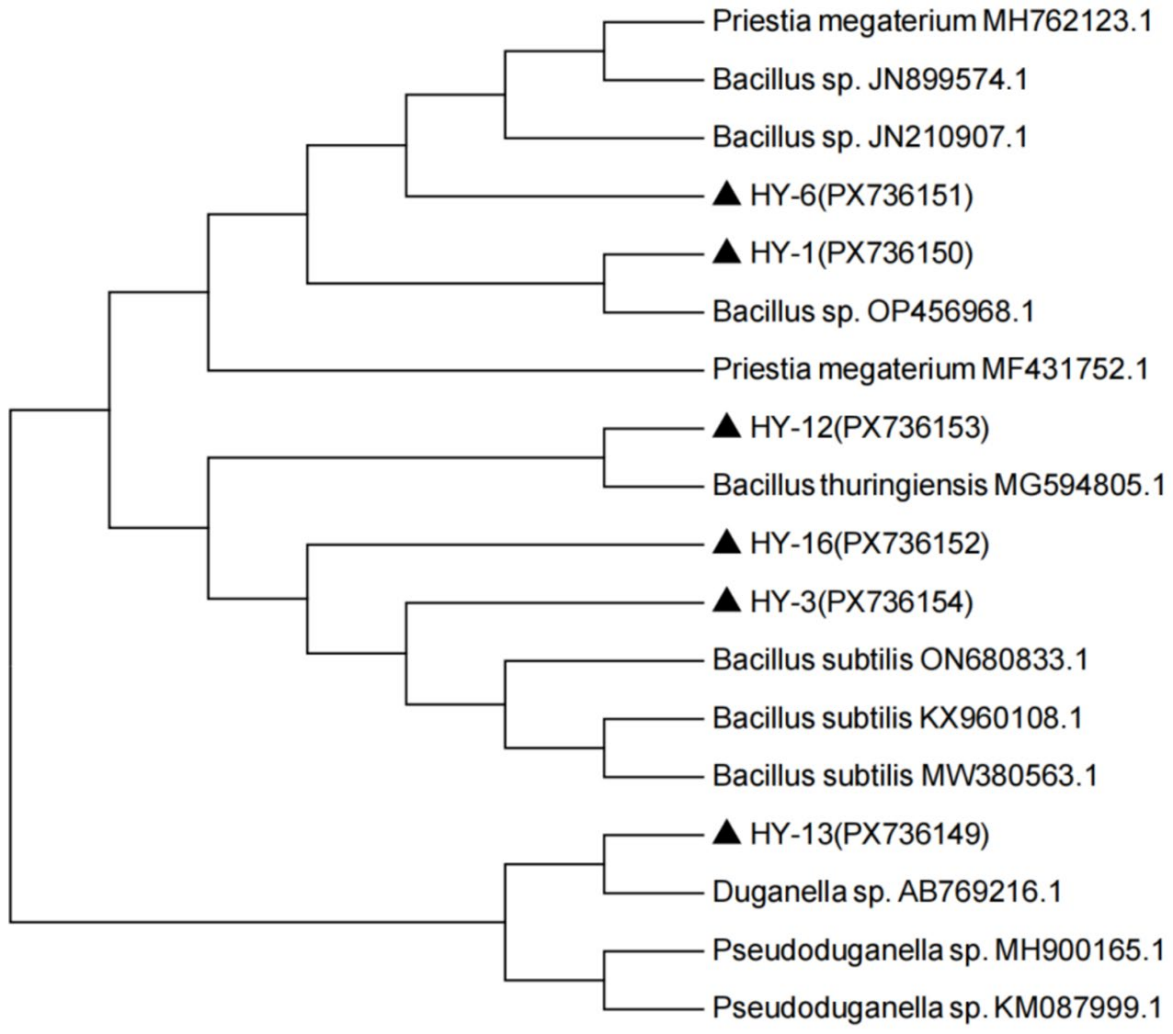
Neighbor-joining phylogenetic tree based on 16S rDNA sequences of PSB strains HY-1, HY-3, HY-6, HY-12, HY-13 and HY-16 and their closest relatives. The tree was constructed in MEGA 7.0 using reference sequences retrieved from GenBank; strain designations from this study are shown in bold.

Six PSB strains (HY-1, HY-3, HY-6, HY-12, HY-13 and HY-16) were subjected to 16S rDNA sequencing, and a phylogenetic tree was constructed using MEGA 7.0 (Figure 9). Meanwhile, the obtained sequences were deposited in NCBI GenBank under accession numbers PX736149–PX736154. The results showed that HY-1 and HY-6 clustered with *Bacillus* spp., HY-3 and HY-16 were most closely related to *Bacillus subtilis*, HY-12 clustered with *Bacillus thuringiensis*, and HY-13 was affiliated with *Duganella* sp.

## 4 Discussion

Microbial metabolic activities profoundly influence the mobility and bioavailability of multiple elements and play a critical role in the biogeochemical cycling of both heavy metals and macronutrients (Wan et al., 2020; Qin et al., 2023). PSB with certain levels of heavy metal tolerance can increase the pool of available soil P through metabolic processes, while simultaneously immobilizing or transforming heavy metals (Chen et al., 2023). Currently, the main PSB applied to remediate heavy metal–contaminated soils and promote plant growth belong to genera such as *Bacillus*, *Cronobacter*, *Pseudomonas* and *Enterobacter* (Xu and Chen, 2025). In the present study, 18 PSB isolates with phosphate-solubilizing capacity were obtained from farmland soils in Baitu Town, Gaoyao District, Zhaoqing City, Guangdong Province, China. Long-term soil acidification in this region, where pH values typically range from 4.5 to 5.5, has increased the bioavailability of chromium and lead, primarily in the forms of Cr^3+^ and Pb^2+^. Despite the ecological relevance of such acidic conditions, the adaptive mechanisms employed by bacterial strains in acidified, metal-contaminated soils remain poorly understood. Six isolates exhibited clear tolerance to both Pb and Cr and were identified as *Bacillus* sp. (HY-1, HY-6), *Bacillus subtilis* (HY-3, HY-16), *Bacillus thuringiensis* (HY-12) and *Duganella* sp. (HY-13). Members of the genus *Bacillus* are well recognized for their phosphate-solubilizing capacity and have been widely reported in studies of soil nutrient cycling and bioremediation. In contrast, reports describing phosphate solubilization by *Duganella* species remain relatively limited, as this genus is more commonly associated with nitrogen cycling. Nevertheless, Fang et al. (2019) have also demonstrated that *Duganella* possesses phosphate-solubilizing ability, supporting the functional versatility observed in strain HY-13 in this study. Although the phosphate-solubilizing capacities of these genera have been reported previously, there are relatively few studies on their tolerance to Pb and Cr. Previous studies have shown that heavy metal contamination can increase the relative abundance of *Duganella* species. Spearman’s rank correlation analysis revealed a significant positive correlation between *Duganella* abundance and Zn concentration, indicating a notable tolerance of *Duganella* spp. to zinc stress (Mao et al., 2024). In this study, the selected strains showed high phosphate-solubilizing capacities and good tolerance to Pb and Cr with higher tolerance to Cr than to Pb. Among them, strain HY-1 (*Bacillus* sp.) exhibited the highest metal tolerance, with MIC values of 20 mmol/L for Cr and 7 mmol/L for Pb. These findings are consistent with previous reports. Rubab et al. (2023) showed that *Bacillus* sp. SR18 exhibited a Cr tolerance of up to 20 mmol/L, as determined by MIC assays on nutrient agar, while Pepi et al. (2016) identified a Pb-resistant *Bacillus pumilus* strain isolated from highly polluted marine sediments at the Sarno River mouth in Italy, with a Pb MIC of 4.8 mmol/L. Similarly, Monga et al. (2024) reported that *Bacillus enclensis* displayed substantially higher tolerance to Cr than to Pb. The marked difference in tolerance between these two metals is largely attributable to the more effective detoxification mechanisms available for Cr. These include active efflux mediated by chromate transporters and the enzymatic reduction of Cr(VI) to the less toxic Cr(III) by NAD(P)H-dependent oxidoreductases. In contrast, Pb cannot be enzymatically detoxified and instead binds strongly to negatively charged functional groups on the bacterial cell surface, leading to persistent toxicity and consequently lower Pb(II) tolerance.

In addition, phosphorus metabolism in PSB may indirectly contribute to enhanced Cr tolerance. In the present study, the selected strains maintained measurable phosphate-solubilizing activity under Cr stress; for example, *Bacillus thuringiensis* HY-12 produced 91.02 mg/L soluble P at 3 mmol/L Cr. The released phosphate ions can subsequently react with Cr^3+^ to form insoluble chromium phosphate precipitates, thereby decreasing the concentration of bioavailable Cr^3+^ through biomineralization. Although Pb^2+^ can similarly form insoluble lead phosphate, such precipitates are less stable under acidic conditions and tend to redissolve, resulting in renewed Pb^2+^ release and sustained toxicity (Teng et al., 2019). This mechanism provides a plausible explanation for the stronger growth inhibition observed under Pb stress than under equivalent Cr concentrations; for instance, *Bacillus subtilis* HY-16 exhibited a 62.3% reduction in OD₆₀₀ at 6 mmol/L Pb, compared with only a 38.5% decrease under the same Cr concentration.

Further analysis of the effects of Pb and Cr on the growth and metabolism of the six strains revealed that both growth and phosphate-solubilizing activity were inhibited to varying degrees under metal stress. Under gradient Cr stress at concentrations of 3, 7 and 11 mmol/L, both bacterial growth and phosphate-solubilizing capacity of all PSB strains declined significantly with increasing Cr levels. This inhibitory effect was most pronounced at the highest concentration tested (11 mmol/L), where the minimum OD₆₀₀ value of 0.289 was observed for strain HY-16. In addition, OD₆₀₀ values were positively correlated with soluble phosphate concentrations, whereas both parameters showed significant negative correlations with Pb and Cr concentrations. Gupta et al. (2018) isolated a *Klebsiella* strain CPSB4 from Cr-contaminated farmland soil, which produced clear phosphate-solubilizing halos at different Cr concentrations. Although its plant growth–promoting activity gradually decreased as Cr concentrations increased to 200 mg/L, it was not completely lost. One possible explanation is that soluble phosphate released into the medium reacts with Pb^2+^ or Cr species to form insoluble metal phosphates, thereby reducing the concentration of soluble phosphate in the culture (Li et al., 2022). In a metagenomic study on PSB harboring the *phoD* or *pqqE* genes in soils with different levels of combined heavy metal contamination, the relative abundance of PSB decreased as total heavy metal concentrations increased, and members of the phylum *Nitrospirae* were particularly sensitive to metals such as Cr and Pb (Mi et al., 2025). In heavy metal–contaminated environments, PSB are subjected not only to direct toxicity from metals but also to secondary stress arising from P limitation, and the combined effects of these pressures can further impair microbial ecological functions.

The phosphate-solubilizing performance of PSB is influenced by both intrinsic microbial traits and environmental factors. Their solubilization capacity directly determines their potential effectiveness for remediating heavy metal–contaminated soils, because stronger phosphate solubilization can release more phosphate and reduce heavy metal mobility and bioavailability (Zhang et al., 2023; Fan et al., 2024). To better exploit the application potential of the six PSB strains, we used single-factor experiments to optimize their fermentation conditions. The results showed that glucose was the optimal carbon source for all six strains, consistent with the findings reported by Han et al. It is proposed that pyruvate generated during glucose metabolism serves as a central metabolic precursor for both organic acid production, which is critical for phosphate solubilization, and the synthesis of metal-binding proteins involved in heavy metal tolerance. This metabolic linkage provides new insight into the synergistic relationship between phosphate solubilization and heavy metal resistance in phosphate-solubilizing bacteria. pH is one of the key factors controlling P availability. In the present study, five strains exhibited the highest growth and phosphate-solubilizing activity at pH 6, whereas growth and phosphate solubilization were inhibited as pH increased beyond this value. This is likely because one of the main phosphate-solubilizing mechanisms of PSB is the secretion of organic acids, which chelate metal ions and release phosphate. Elevated pH may reduce organic acid production or alter their dissociation and chelation, thereby decreasing the release of bioavailable phosphate. Additionally, due to the acidic soil environment and microbial niche effect, these microbes have evolved over the long term to be more adapted to acidic conditions, thereby exerting optimal functions. At pH 5, phosphate-solubilizing capacities were also relatively low, possibly because excessive accumulation of organic acids and other acidic metabolites during fermentation lowered the pH to levels unfavorable for bacterial growth. These observations are partially supported by the study of Fitriatin et al. (2021), who examined two *Burkholderia* strains and showed that phosphate-solubilizing bacteria produced higher levels of organic acids and released more soluble P at pH 4.5 than at pH 7 or 10.5. This finding confirms that moderately acidic conditions generally favor PSB-mediated phosphate solubilization, whereas deviations from the optimal pH, either toward alkalinity or excessive acidity, can inhibit this process. All six strains grew best and exhibited the highest phosphate-solubilizing activity at 0.3 g/L NaCl, whereas further increases in NaCl concentration inhibited growth and phosphate solubilization. Collectively, these findings demonstrate the dual functional advantages of the six isolated strains, namely high phosphate-solubilizing efficiency and strong tolerance to Pb and Cr, under acidified and heavy metal–contaminated conditions. The results further elucidate the underlying mechanisms, including Cr-specific detoxification and P metabolism–mediated biomineralization, and define optimized culture conditions. Together, these insights provide a solid foundation for the application of these strains in the bioremediation of Pb- and Cr-contaminated agricultural soils, particularly in acidified regions such as southern China.

## 5 Conclusion

In this study, 18 PSB strains with phosphate-solubilizing ability were isolated from crop rhizosphere soils collected from Baitu Town, Gaoyao District, Zhaoqing City, Guangdong Province, China. All strains solubilized tricalcium phosphate and ferric phosphate, with the highest activity observed for tricalcium phosphate (2.82–253.53 mg/L), and their phosphate-solubilizing capacities followed the order tricalcium phosphate > ferric phosphate > aluminum phosphate > lecithin. Six strains exhibited good tolerance to Pb and Cr, and their growth and phosphate-solubilizing capacities decreased with increasing metal concentrations. Single-factor optimization revealed that glucose was the optimal carbon source, the optimal NaCl concentration was 0.3 g/L, five strains had an optimal pH of 6 (HY-12 showed optimal solubilization at pH 7), and some strains were thermotolerant with maximal phosphate solubilization at 40 °C, whereas others showed optimal activity at 30 °C. Taken together, these six PSB strains combine high phosphate-solubilizing efficiency with strong environmental adaptability, facilitating the release of available soil P and the reduction of heavy metal bioavailability. They therefore represent promising microbial resources and provide a theoretical basis for the remediation of Pb- and Cr-contaminated soils and the development of green agriculture.

## Data Availability

All data generated or analyzed in this study are contained within this published article. The sequence of the strain investigated in this study is publicly accessible via the NCBI database, and its accession numbers are PX736149–PX736154.
